# MicroRNA profiling in serum and humor vitreous of patients affected by vitreoretinal diseases

**DOI:** 10.1186/s40942-025-00765-3

**Published:** 2025-12-23

**Authors:** Maria Ludovica Ruggeri, Erica Costantini, Rossella D’Aloisio, Alberto Quarta, Cecilia Contardi, Agbeanda Aharrh-Gnama, Matteo Gironi, Lisa Aielli, Marta Di Nicola, Marcella Reale, Lisa Toto, Rodolfo Mastropasqua

**Affiliations:** 1 Department of Neuroscience, Imaging and Clinical Sciences, University G. D’Annunzio Chieti-Pescara, Via dei Vestini 31, 66100 Chieti, Italy; 2 Department of Medicine and Science of Ageing, University G. D’Annunzio Chieti-Pescara, Via dei Vestini 31, 66100 Chieti, Italy; 3 Ophthalmology Clinic, Department of Medicine and Science of Ageing, University G. D’Annunzio Chieti-Pescara, Via dei Vestini 31, 66100 Chieti, Italy; 4 Department of Innovative Technologies in Medicine and Dentistry, University G. D’Annunzio Chieti-Pescara, Via dei Vestini 31, 66100 Chieti, Italy; 5 Laboratory of Biostatistics, Department of Medical, Oral and Biotechnological Sciences, “G. d’Annunzio” University of Chieti-Pescara, 66100 Chieti, Italy

**Keywords:** Retinal detachment, Vitreomacular diseases, MicroRNA, OCT angiography, Biomarkers

## Abstract

**Background:**

To analyze the differential expression of microRNAs (miRNAs) in humor vitreous and serum of patients affected by vitreomacular diseases (VMD) and rhegmatogeneous retinal detachment (RRD). Secondly, to assess their correlation with perfusion parameters during post-operative follow-ups.

**Methods:**

Patients diagnosed with vitreomacular interface disease (VMD) and rhegmatogeneous retinal detachment (RRD) scheduled for pars plana vitrectomy (PPV) were included in our prospective interventional study. All patients underwent multimodal imaging including optical coherence tomography angiography (OCTa) and vitreous humor and serum sampling.

**Results:**

A total of 38 participants were enrolled. Among them, 19 (50%) were diagnosed with VMD and 19 (50%) with macula on- RRD. Among the 105 commonly expressed sequences, six miRNAs (hsa-miR-21-5p, hsa-miR-222-3p, hsa-miR-451a, hsa-miR-146a, hsa-miR-144-3p and hsa-miR-320a) were significantly upregulated, despite differences in folds between the vitreous humor and serum levels. In both groups of diseases, hsa-miR-320a showed a significant correlation with the superficial capillary plexus (SCP) macular vessel density in RRD (rho = 0.499) and iVMD (rho = 0.636) while hsa-miR-144-3p exhibited a negative correlation (rho = −0.776) with the SCP variation in the iVMD group.

**Conclusion:**

The differential expression of miRNAs in patients affected by VMD and RRD, alongside their correlation with perfusion parameters, supports miRNA application in clinical practice as valuable biomarkers for their potential predictive role.

**Supplementary Information:**

The online version contains supplementary material available at 10.1186/s40942-025-00765-3.

## Introduction

Vitreomacular interface disease (VMD) and rhegmatogenous retinal detachment (RRD) are two of the most common indications in vitreoretinal surgery, which significantly affect the general population [1, 2]. Such procedures not only require achieving the surgical goal but also necessitate strict follow-up examinations to ensure a positive outcome. In this context, biomarkers serve as valuable tools for monitoring the patient’s disease progression and, ideally, predicting the final outcome. While Optical coherence tomography (OCT) and Optical coherence tomography angiography (OCTa) have widely been acknowledged as useful tools in the patient management, today their predictive role has not been overall accepted [3, 4].

Nevertheless, dynamic microvascular changes have previously been detected in patients affected by vitreomacular diseases (VMD) pointing out vascular modifications that can occur in newly established post-operative connections [5].

MicroRNAs (miRNAs) are non-coding transcriptional RNA products with a central role in regulating gene expression in proliferation, angiogenesis, apoptosis, immunity, and other biological processes [6, 7].

Studies have reported their dysregulation in human tissues affected by cancer, inflammation, diabetes, cardiovascular and neurological diseases, alongside their expression in biological fluids including vitreous humor, thus opening to their possible role as predictive biomarkers [8–11].

Several authors have previously shed light on miRNA levels deregulation in Ophthalmology. Usui-Ouchi et al. found upregulation of hsa-miR-21 in the vitreous in proliferative vitreoretinal diseases including proliferative diabetic retinopathy (PDR), and proliferative vitreoretinopathy (PVR) [12].

Likewise, Tuo et al. found hsa-miR-155 differently expressed in the case of primary vitreoretinal lymphoma (PVRL), thus helping in the differential diagnosis with uveitis [13]. Consistently, Chadalawada et al. described the miRNAs expression signature in the vitreous of patients affected by intraocular tuberculosis. Moreover, the study of miRNAs has brought promising results in age-related macular degeneration (AMD), opening to their possible role as key factors for a better comprehension of posterior segment diseases [14].

However, there is a lack of extensive literature about the role of miRNA in vitreoretinal diseases, although their application may be useful to monitor and predict diseases [15].

In fact, although different conditions, iVMD and RRD share in common the posterior segment involvement and the surgical management, which alongside their high incidence suggests the necessity of tools able to assess and predict surgical success.

A recent review by Jiang et al. has studied the role of miRNA in retinal Müller glial cell function, offering interesting insights into miRNA involvement in the process of neuroprotection, regeneration, inflammation, and development of retinal disease, pointing out miRNAs in Müller glial cells as a potential diagnostic and therapeutic target in retina repair and regeneration [16].

To obtain surgical success, structural anatomical and neurovascular integrity is necessary, thus recalling reparation and remodelling processes. Nevertheless, it is not clear whether miRNA may be related to vascular changes of newly reestablished connection, nor if their level could be used as follow-up tools. Therefore, the aim of our study was to investigate the vitreal miRNA expression in comparison with serum miRNA in patients with iVMD and RRD and to explore their correlation with post-operative perfusion changes.

## Materials and methods

### Patients’ characteristics

A total of 38 eyes of 38 subjects were enrolled in our prospective interventional study. All patients were enrolled at the Ophthalmology Clinic of University “G. d’Annunzio”, Chieti-Pescara, Italy. 19 eyes were diagnosed with iVMD (MHs and ERMs), whereas 19 patients were diagnosed with RRD. All patients were candidates for surgery.

Inclusion criteria were: (1) idiopathic ERMs at stage 3 or idiopathic MHs at stage 4 or recent onset macula-ON RRD, defined as visual symptoms within three days, not extending beyond vascular arcades; (2) ERMs duration ≤ 6 years or MH duration ≤ 1 year

Exclusion criteria were: (1) history of previous ocular surgery; (2) evidence or history of ocular conditions other than ERMs, MHs or macula on RRD; (3) macular hole RD (4) High myopia, which was defined as spherical equivalent (SE) of ≤ −6 diopters (D) or axial length (AL) of ≥26.5 mm (5) evidence or history of systemic disorders, including diabetes and systemic hypertension; (6) poor image quality or no patient’s collaboration; (7) glaucoma; (8) blood hypertension; (9) diabetes mellitus; (10) blood hypercoagulability conditions. The study was approved by the institutional review board and adhered to the tenets of the Declaration of Helsinki (code RNAVTR, n. 226/9.02.2022). Informed consent was obtained from all participants.

### Ophthalmic procedures

All patients underwent complete ophthalmological examination including best corrected visual acuity (BCVA) expressed in logMAR, intraocular pressure measurement (IOP) with Goldmann applanation tonometry, fundus examination with indirect ophthalmoscopy after 1% tropicamide instillation. All examinations were performed at baseline and repeated three and six months after surgery. Moreover, SS-OCTA and ultra-wide field retinography (UWF) were performed at each visit from baseline. Partial coherence laser interferometry measured axial length (IOL Master 700; Carl Zeiss Meditec, Jena, Germany). All images were acquired by a single trained ophthalmologist (AQ).

Images were analyzed by two different ophthalmologist experts in the retina field (LT and RM). ERMs were classified according to Govetto staging system whereas MHs were classified according to Gass et al. [17, 18].

**Fig. 1:**
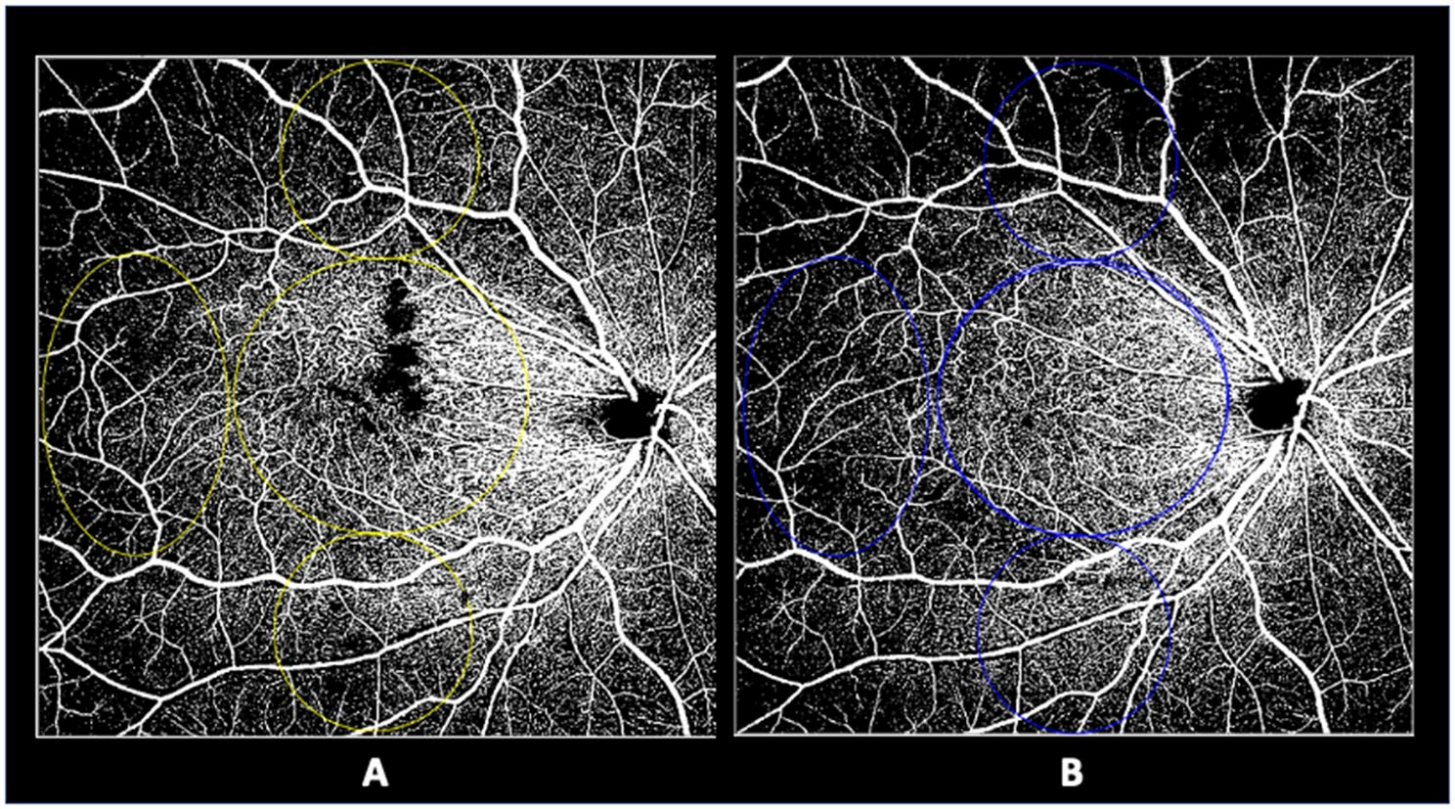
Superficial capillary plexus (SCP) slab identified on optical coherence tomography (OCTA) analysis before (**A**) and after (**B**) surgery for epiretinal membrane showing reperfusion. The quantitative analysis was performed after binarization and skeletonization through analysis around the fovea and extended to the mid-peripheral area after identification of three different locations by three circles (temporal, superior, and inferior)

Ultra-widefield retinography (Optos California, Optos, Inc. Marlborough, MA, USA) was performed at each gaze position. All patients underwent SS-OCTA (Plex Elite 9000; Carl Zeiss Meditec, Inc.) with a 12 mm × 12 mm scan protocol. Only images with a signal strength index > 8 were accepted and the software segmentation tool was applied in all cases to identify the superficial capillary plexus (SCP) and deep capillary plexus (DCP) slabs. Images were imported and thus analyzed into ImageJ, *ImageJ software version 1.50* (National Institutes of Health, Bethesda, MD; available at https://imagej.net/ij/index.html) and then processed to be binarized and thus skeletonized, as previously described [19]. The quantitative analysis was performed in the macular region, which was defined as a circular annulus around the fovea with a diameter of 4 mm excluding the foveal avascular zone (FAZ) and in the mid periphery three circles (temporal, superior, and inferior of 3 mm) as previously reported (Fig. 1) [19].

### Surgical procedures

All patients underwent a three-port 25-G pars plana vitrectomy with Constellation (Alcon Laboratories Inc, Fort Worth, TX) under retrobulbar anestesia and were performed by a single expert vitreoretinal surgeon (RM). In the case of phakic patients, a combined procedure was performed. All the procedures followed.

After vitreous sample collection, the presence of posterior vitreous detachment (PVD) was checked with triamcinolone acetonide (Vitreal S, Fidia farmaceutici, Italy). A vitrectomy was thus completed with careful peripheral check. The following steps were performed according to the dedicated procedure.

#### ERM surgery

ERM/ILM were stained with 0.3-mL intravitreal dye Membrane Blue-Dual (DORC International, Zuidland, the Netherlands; 0.15% trypan blue, 0.025% Brilliant Blue G). Through the help of a fine-tipped forceps (Alcon ILM forceps 25 G) the peeling of the stained membranes was performed up to the vascular arcades, either with or without ILM peeling based on the surgeon’s preferences. A tamponade agent (air, fluid, or sulfur hexafluoride) was used to fill the vitreous cavity. All patients received a subconjunctival injection of anti-inflammatory and antibiotics as standard protocol at the end of surgery.

#### MH surgery

Brilliant Blue G (ILM Blue; Dorc International, Zuidland, Netherlands) was used to stain the internal limiting membrane, which was peeled using a pinch technique and end-gripping forceps (Grieshaber Revolution DSP ILM Forceps; Alcon Grieshaber AG, Schaffhausen, Switzerland).

A tamponade agent (20% sulfur hexafluoride) was used, and patients were instructed to follow a face-down position for 3 days after surgery.

#### RRD surgery

A flute needle was used to aspirate subretinal fluid, and any retinal breaks were photocoagulated using endolaser. Air-gas exchange with diluted gas as tamponade, SF6 (20%) or C3F8 (12%), was performed, and the sclerotomy was sutured when required. Patients were told to stay face down for one to three days.

### Vitreous and serum sample collection

Undiluted 1 mL vitreous sample was aspirated via pars plana with a 25 gauge vitreous cutter at the beginning of vitrectomy before the infusion port opening. The vitreous cutter was set at high cut rates (≥7500 cuts per minute) to obtain a sample connected to a 1 mL syringe. The instrument was carefully maneuvered to obtain a mid-vitreous sample and moved to different areas with care. Eye volume was maintained by simultaneous air exchange while the sample was removed to avoid pre-incisional hypotony. No complications occurred during the procedure.

Dry vacutainer tubes collected serum from blood samples drawn via vein puncture (BD Biosciences, Italy). Clotting was assured by keeping the samples at room temperature for at least 30 min. The samples were centrifuged for serum isolation at 7000 rpm for 15′ at 4 °C. The obtained samples were immediately frozen in aliquots in a polypropylene tube at −80 °C until assay.

### MiRNA isolation and reverse transcription from vitreous humor and serum

Vitreous humor and serum samples were centrifuged at 3000 rpm for 5’ at 4 °C to eliminate debris. The supernatant was then used for the subsequent miRNA extraction. Using the miRNeasy Advanced Kit (Qiagen, Hilden, Germany), the total RNA, including miRNA, was isolated from 200 µl of each sample. The RNA was quantified and quality checked on NanoDrop™ 2000/2000c (Thermo Scientific, Waltham, Massachusetts, USA). An acceptable RNA purity was defined with a ratio OD260/OD280 > 1.8 and < 2.1.

Reverse transcription reaction for first-strand cDNA was performed using the miRCURY LNA-RT Kit (Qiagen, Hilden, Germany) on 5 ng of each sample following the manufacturer’s protocol.

### MicroRNA expression profiling

Keeping differentiated biological specimens and disease types (vitreous humor of iVMD; serum of iVMD; vitreous humor of RRD and serum of RRD), 3 µl of the synthesized cDNA from each sample was pooled for the miRNAs screening analysis.

The miRCURY LNA™ miRNA Focus PCR Panel was applied to profile the expression of 191 miRNAs in the vitreous humor and serum samples. Then, using CFX Real-Time PCR Detection Systems (Bio-Rad, Hercules, California, USA) and the following amplification program (defined as 95 °C for 10‘, followed by 40 cycles of 95 °C for 15’ and 60 °C for 1’), the reaction mix, for each study group, was analyzed by quantitative polymerase chain reaction (qPCR).

In the study’s second phase, selected miRNAs were analyzed in each sample (n.19 iVMD and n.19 RRD). Specific miRNA sequences chosen after the screening phase, were evaluated with miRCURY LNA SYBR® PCR kit. Each sample will be run in triplicate. miRNAs showing a maximum Ct = 35 will be included in the analysis based on the average cycle threshold (Ct) to apply the the 2^-ΔΔDCt^ method and to select the reference gene [20].

### Statistical analysis

For each group and each miRNA sequence, 5° and 95° percentile of Ct distribution of genes were calculated. Genes with values ≤ 5° percentile or ≥ 95° percentile were selected. Descriptive statistics are expressed by median, 1st and 3rd quartile [q1; q3]. Normality distribution was verified by the Shapiro-Wilk test. Spearman rho correlation coefficient was used to reveal associations between has-miRNAs and also perfusion parameters, and a statistically significant relationship was reported.

The Wilcoxon Rank-Sum test was used to assess longitudinal variations in clinical and perfusion parameters (BCVA, OCT and OCTA indices) between baseline and the 6-month follow-up. miRNA expression data were obtained at the time of surgery only, as post-operative vitreous sampling was not ethically feasible.

Between groups differences were assessed by the Mann U Whitney test. Spearman’s rank correlation coefficient (rho) was used to identify potential correlations between the hsa-miRNAs expression levels and retinal perfusion parameters, including vessel density (VD) of the superficial and deep capillary plexus (SCP and DCP) in the macular, superior, inferior, and temporal regions, as well as central macular thickness (CMT) and subfoveal choroidal thickness (SFCT), revealing statistically significant associations.

All the tests were two-sided, and a level of statistical significance was set at *p* ≤ 0.05. All the statistical analyses were performed using the R software environment for statistical computing and graphics version 4.3 (R Foundation for Statistical Computing, Vienna, Austria. https://www.R-project.org/).

## Results

The study cohort had a mean age of 63.5 years (standard deviation = 8.53), with males representing the majority (26 of 38 participants; 68%).

### MiRNA screening for differential expression

**Fig. 2:**
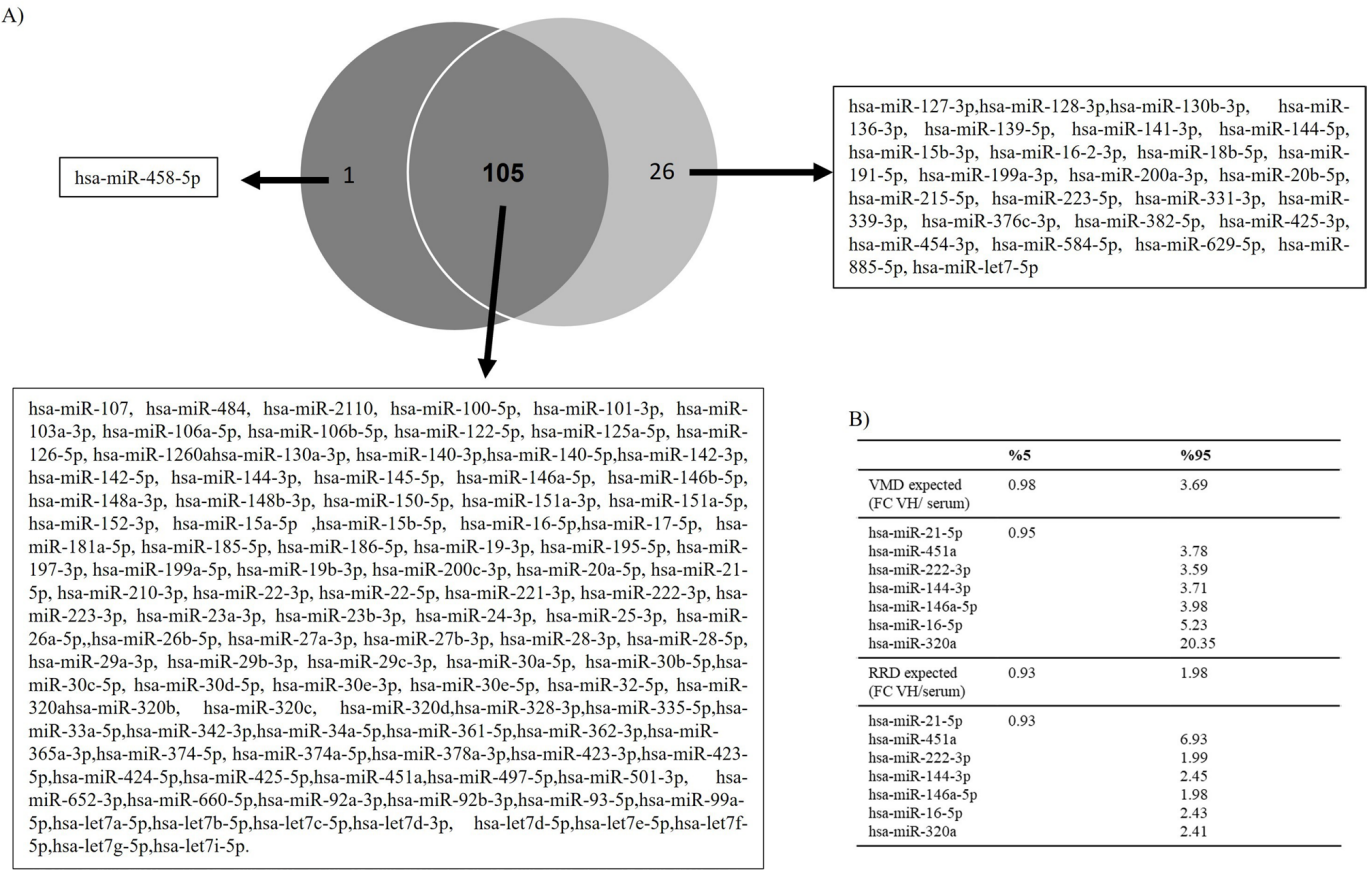
(**A**) Venn’s diagram depicts the proportion of miRnas consistently dysregulated in vitreous humor and serum samples. (**B**) Table with 5th and 95th percentiles for selection of main interesting miRNA

A total of 191 miRNAs were screened. Among them, 105 sequences were found to be commonly expressed in both serum and vitreous humor of patients affected by iVMD or RRD (Fig. 2).

Among the 105 commonly expressed sequences, six miRNAs (hsa-miR-222-3p, hsa-miR-451a, hsa-miR-146a, hsa-miR-144-3p and hsa-miR-320a) were significantly upregulated and one miRNA (hsa-miR-21-5p) showed only a slight reduction to the 5° percentile. These seven miRNAs were selected for the following validation step in each patient.

### Comparison of miRNAs expression in vitreous and serum

The miRNA assay for hsa-miR-222-3p, hsa-miR-451a, hsa-miR-146a-5p, hsa-miR-144-3p, hsa-miR-320a and hsa-miR-21-5p, were performed in all 38 patients, both in vitreous humor and serum using hsa-miR-16-5p as reference gene.

**Fig. 3:**
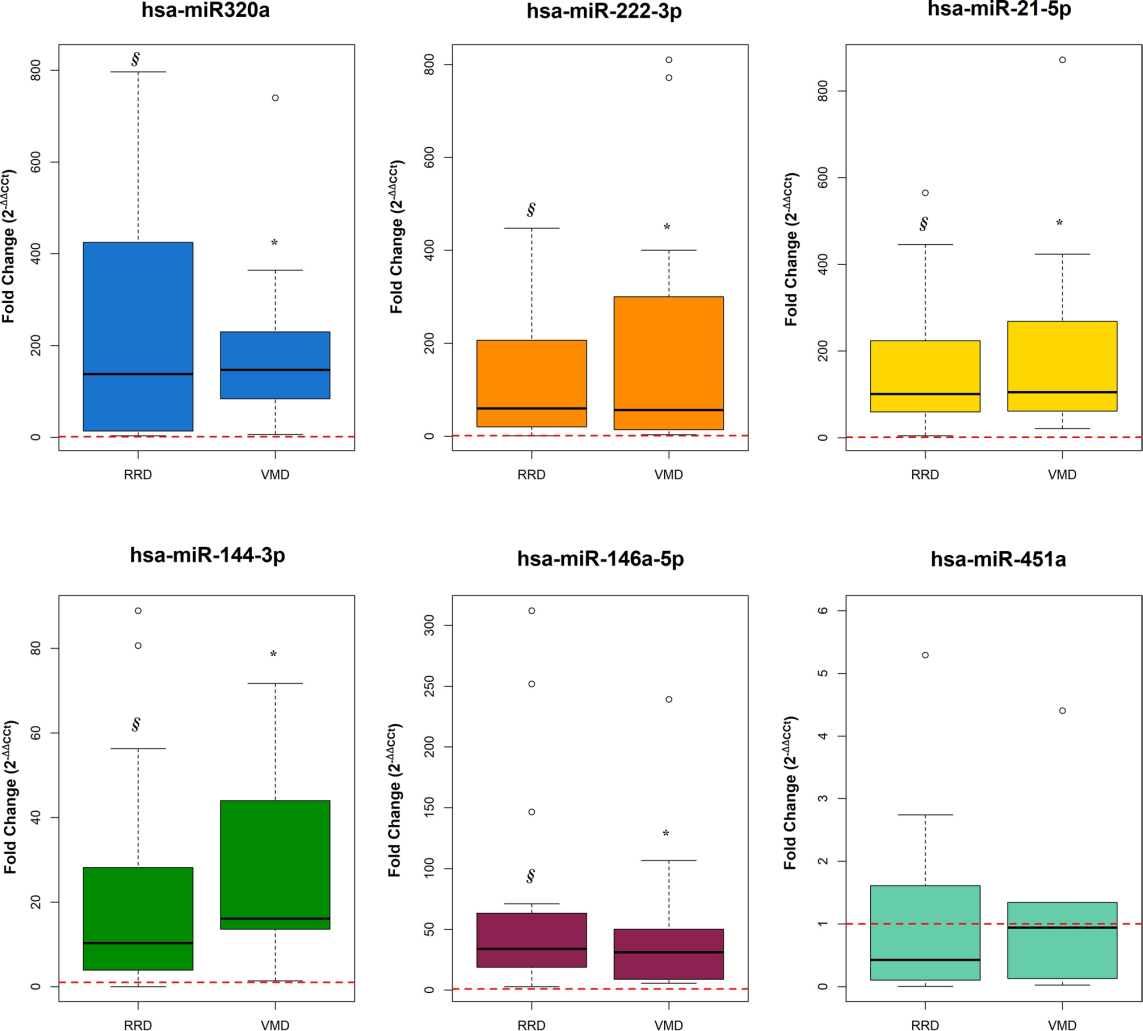
Box and whiskers plot of the Fold changes of miRnas in humor vitreous relative to serum, in iVMD and RRD eyes. miRnas expression in humor vitreous in comparison to serum of hsa-miR-222-3p (iVMD 56.4 FC; RRD 59.9 FC); hsa-miR-146a-5p (iVMD 31.2 FC; RRD 30.9 FC); hsa-miR-144-3p (iVMD 16.1 FC; RRD 11.4 FC); hsa-miR-320a (iVMD 147 FC; RRD 138 FC); hsa-miR-21-5p (iVMD 105 FC; RRD 105 FC) and hsa-miR-451a (iVMD 0.94 FC; RRD 0.43 FC) showed statistical differences *p < 0.05 humor vitreous vs serum in iVDM; ᵴ *p* < 0.05 humor vitreous vs serum in RRD

As stated, miRNAs expression analysis in iVMD and RRD demonstrated the commonly expression of the selected miRNAs. The fold differences of miRNAs in the vitreous humor to serum were statistically significantly higher both in iVMD and RRD groups. In contrast, the data on hsa-miR-451a showed a down-regulation, although not statistically significant differences between the two groups (0.94 [0.13;1.34] and 0.43 [0.11;1.52] in the VMD and RRD group respectively, *p* = 0.810) (Fig. 3, Table 1, Supplementary table 1).

**Table 1:** FC values in the VMD and RRD group. (VMD: vitreomacular disease; RRD: rhegmatogeneous retinal detachment)

**2**^-ΔΔ**Ct** (^**Vitreous vs. Serum)**	VMD	RRD	p-value
hs-miR320a	147 [83.9;230]	138 [16.8;377]	0.873
hs-miR21	105 [61.5;268]	101 [61.6;219]	0.749
hs-miR222	56.4 [14.0;300]	59.9 [20.7;187]	0.841
hs-miR144	16.1 [13.6;44.0]	11.4 [6.94;45.4]	0.662
hs-miR146	31.2 [8.91;50.2]	30.9 [17.9;61.4]	0.957
hs-miR451	0.94 [0.13;1.34]	0.43 [0.11;1.52]	0.810

Median [q1 = first; q3 = third] quartile for miRs fold in VMD and RRD group. *P*-value derives from the Mann U Whitney test

Table Median [q1 = first; q3 = third] quartile for miRs fold in VMD and RRD group P-value derives from the Mann U Whitney test.

#### Functional parameters

Clinical data were collected at baseline and six months after surgery. At the six-month follow-up, an increase in BCVA was observed in both groups (*p* = 0.060). SCP and DCP slabs showed reperfusion with a significant increase in macular vessel density assessed during the two groups’ follow-up (Table 2). No PVR, ERM MH recurrence or other forms of anatomical failure was reported (*n* = 0).

**Table 2:** Descriptive statistics of perfusion parameters by disease group expressed as median [q1; q3] and absolute difference (∆) between T1 = 6 months follow-up and T0= baseline

Variables	VMD				RRD			
	T0	T1	∆ (T1–T0)	p-value	T0	T1	∆ (T1–T0)	p-value
BCVA (LogMAR)	0.52 [0.40;1.52]	0.30 [0.10;0.40]	−0.40 [−1.20; −0.10]	**0.001**	0.15 [0.00;0.40]	0.05 [0.00;0.06]	−0.10 [−0.31;0.00]	0.060
SCP MACULAR VD	27.6 [23.0;37.6]	35.1 [30.0;49.9]	7.11 [2.66;12.8]	**0.040**	30.2 [26.5;39.2]	39.7 [32.7;42.4]	4.86 [−7.90;16.0]	**0.025**
SCP SUPERIOR VD	26.2 [23.4;31.0]	22.4 [18.6;30.4]	−1.78 [−7.62;3.55]	0.642	31.0 [21.9;37.0]	27.9 [24.1;33.5]	−0.89 [−10.65;6.85]	0.417
SCP INFERIOR VD	28.8 [27.9;30.9]	27.6 [24.3;29.2]	−0.86 [−5.93;1.29]	0.278	32.6 [16.3;38.5]	32.9 [26.6;37.0]	3.39 [−5.17;8.97]	0.871
SCP TEMPORAL VD	22.6 [19.9;23.8]	20.8 [19.0;25.0]	−1.19 [−7.38;4.51]	0.617	21.3 [14.8;27.7]	21.0 [15.1;24.7]	0.01 [−11.49;5.67]	0.646
DCP MACULAR VD	31.6 [25.2;35.9]	30.9 [24.3;37.0]	−2.23 [−9.71;6.97]	0.796	38.5 [33.4;45.0]	44.3 [40.6;47.7]	7.22 [−4.77;15.0]	**0.030**
DCP SUPERIOR VD	26.0 [22.9;31.8]	28.1 [24.0;30.2]	2.10 [−6.24;6.55]	0.730	26.3 [23.2;30.5]	26.5 [19.3;32.7]	2.46 [−6.49;8.35]	1.000
DCP INFERIOR VD	32.0 [29.1;35.3]	29.9 [23.1;33.7]	−3.04 [−11.17;4.57]	0.380	31.1 [16.6;36.5]	26.4 [22.1;31.6]	−0.34 [−10.97;7.08]	0.598
DCP TEMPORAL VD	28.8 [19.0;31.0]	25.5 [17.7;54.5]	0.64 [−7.19;15.8]	0.459	24.4 [18.5;29.4]	18.9 [16.9;25.0]	−2.16 [−11.92;8.04]	0.267
CMT (μm)	507 [220;527]	480 [197;495]	−31.00 [−42.00; −20.00]	**0.044**	232 [225;238]	221 [210;238]	−11.00 [−20.50;5.25]	0.103
SFCT (μm)	205 [196;210]	190 [190;206]	−12.00 [−20.00;7.00]	0.261	220 [214;235]	214 [205;226]	−3.50 [−28.25;9.75]	0.159

*P*-value derived from Wilcoxon rank-sum test. BCVA: best corrected visual Acuity; SCP: superficial capillary Plexus; DCP: deep capillary Plexus; VD: vessel Density; CMT: central macular Thickness; SFCT: subfoveal choroidal thickness

### Correlation of miRNAs and retinal perfusion parameters

**Fig. 4:**
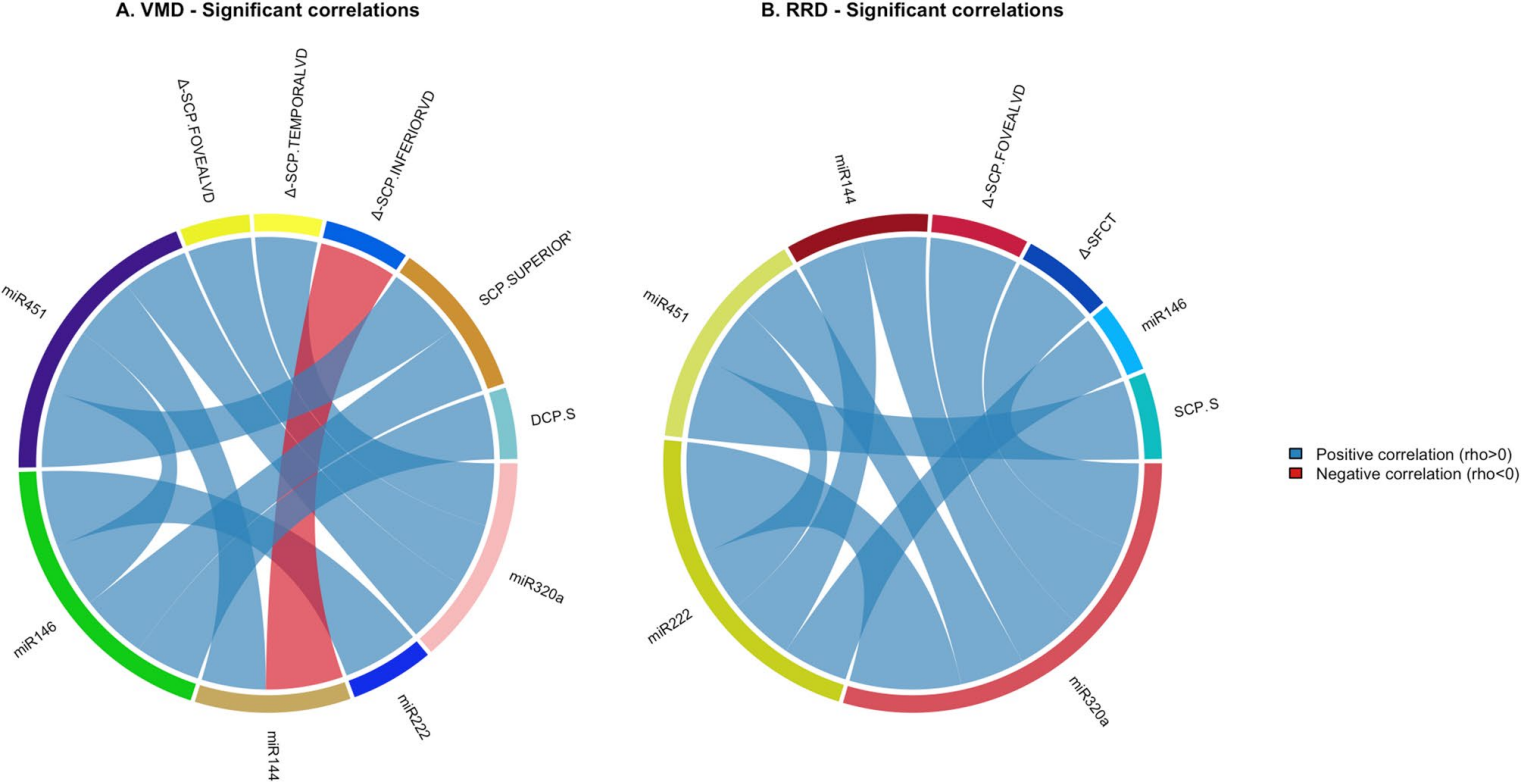
Correlation network between expression levels of miRnas and pefusion parameters using as ties the Spearman rank correlation coefficient that result to be statistically significant for VMD and RRD group

Figure 4 shows the correlation network analysis revealed disease-specific relationships between miRNAs expression and perfusion parameters in each group (based on the Spearman correlation coefficient). Only statistically significant correlations are showed. Notably, hsa-miR-320a showed opposite correlations with SCP macular vessel density variation, being positively associated in iVMD (rho = 0.636; *p* = 0.022) and negatively correlated in RRD (rho = 0.499; *p* = 0.035). This finding suggests that hsa-miR-320a may play divergent roles in the regulation of microvascular remodeling depending on the underlying retinal condition. Conversely, hsa-miR-144-3p displayed a consistent negative correlation (rho = −0.776; *p* = 0.011) with SCP inferior vessel density in iVMD, potentially reflecting its involvement in oxidative stress and reperfusion processes.

### Interactive miRNA target gene and target pathway networks analysis

**Fig. 5:**
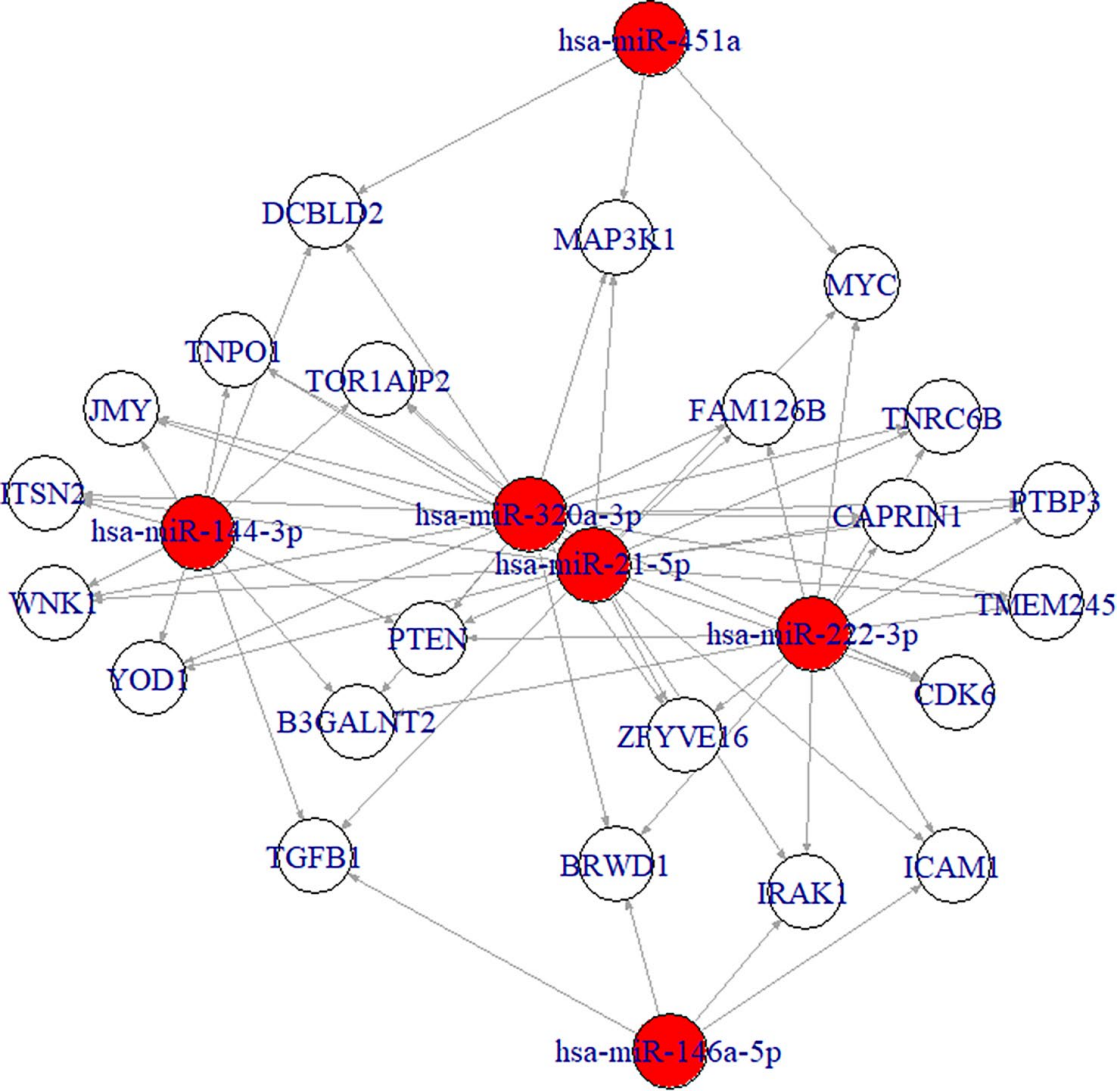
Graph of miRnas target analysis. miRtargetlink 2.0 analysis of putative targets was performed for the six miRnas evaluated in VMD and RRD eyes. In red round are highlighted the miRnas code and in white round there are the target

Analyzing the putative target genes and target pathways is essential in noncoding RNA studies. In our systems biology context, we used miRTargetLink 2.0 (https://ccb-compute.cs.uni-saarland.de/mirtargetlink2/), an interactive tool for the preliminary hierarchical description of miRNA regulatory networks containing validated and predicted target genes. As reported in Fig. 5, the selected miRNAs can be related to the regulation of inflammatory mediators through the nuclear factor-kappa B (NFkB) pathway (hsa-miR-222-3p, hsa-miR-146a-5p, hsa-miR-21-5p), to the cell invasiveness and angiogenesis (hsa-miR-21-5p, hsa-miR-222-3p, hsa-miR-451a, hsa-miR320a-3p) and to the regulation of the antioxidant activity through the Nuclear factor erythroid 2-related factor 2 (NRF2) signaling (hsa-miR-144-3p, hsa-miR-146a-5p), that are key processes of VR diseases (Fig. 5).

## Discussion

The study of miRNAs has been of interest for years because of their potential use as biomarkers for diagnosing, monitoring, and predicting disease development. As a result, their ability to regulate inflammatory patterns has made them suitable to better understand inflammatory and non-inflammatory disorders. However, in this context, their role in vitreomacular diseases has not been completely explored. Jiang et al., in their recent review, proposed studying miRNA in Müller glial cells as a potential diagnostic and therapeutic tool to enhance retina regeneration and repair processes, which are central in the management of such diseases [16]. In our analysis, specific miRNAs were found to be significantly upregulated in the vitreous of both RDD and iVMD patients when compared to serum levels (Supplemetary Table 2). Specifically, after the miRNA panel analysis, the significant modulation of seven primary miRNAs was found, alongside the upregulation of the hsa-miR-21-5p, hsa-miR-222-3p, hsa-miR-144-3p, hsa-miR-146a-5p, hsa-miR-320a, and the vitreal downregulation of hsa-miR-451a compared to serum samples. These findings demonstrate the possibility of detecting miRNA and cellular activities in the vitreous and using them as biomarkers to study disease onset and evolution. Besides, it is worth noting that these results were obtained from both RRD and VMD patients, thus underlining the common miRNA expression, although physiopathological differences exist in the two conditions.

Previously, a fibrotic potential for hsa-miR-21 has been suggested, as a consequence of high glucose levels and transforming growth factor (TGF)β2 stimulation in retinal pigment epithelial (RPE) cells in PDR and vitreo-proliferative diseases [12].

Moreover, increased hsa-miR-29a-3p and hsa-miR-192-5p expression was found in serum samples of patients with neovascular AMD by Dähmcke and colleagues, with the increase of hsa-miR-335-5p, hsa-miR-192-5p, and hsa-miR-194-5p in serum of patients with PDR whose hsa-miR-100-5p expression was reduced in vitreous samples [21].

Recently, Shanbagh et al. found hsa-miR182-5p to be involved in the regulation of the FoxO1-Akt axis and the upregulation of VEGFR (Vascular Endothelial Growth Factor Receptor) and VEGF (Vascular Endothelial Growth Factor) levels via ZNF24 inhibition, suggesting the possible targeting of miRNAs as a potential treatment in PDR [22]. Due to the miRNA involvement in inflammatory processes, previous attention has also been focused on miRNA potential role in the study of PVR [23–25]. In this setting, it is worth underlining the possible advantages that could derive from their study in PVR prediction at the time of surgery, a factor that, alongside a more effective PVR therapy, could help the surgeons in PVR management and surgery success.

However, we must acknowledge that a single time-point measurement in the context of a dynamic process such as PVR, may be misleading. In this setting, miRNAs could cover their role in the diagnostic process as markers of the current disease state, while recognizing their potential as future prognostic biomarkers.

In this, light, miRNA sequential monitoring would take a part in the postoperative follow-up period, as the kinetics of changes in expression are likely to hold concrete predictive value. Nevertheless, the use as possible biomarkers would require miRNA identification in more accessible compartments to make it more feasible for follow-up studies. As a result, the present findings are currently potentially applicable to RRD rather than PVR in the present form. Moreover, by reflecting the dynamic process of PVR with several time measurements and through validation on a larger cohort, more insights into the miRNA applications in PVR prediction would make it possible their application as follow-up biomarkers.

In this light, we must acknowledge that the only enrollment of macula ON RRD, treated only with gas may represent a limitation due to the low risk of developing PVR.

Differently, other reports have studied miRNA role in RD by highlighting the expression of hs-miR-148a-3p in promoting epithelial-mesenchymal transition in the RPE, while miR-148 vitreal expression has been related to the severity of the RD, in terms of size of the retinal break and disease duration [26, 27].

Differently, other miRNAs have been found to be important in promoting retinal regeneration and injury repair, as demonstrated by Yao et al., who reported miRNAs regulation of let-7 expression by Wnt signaling and Lin28 to influence factors in Muller cells proliferation and conversion [28].

Besides, other miRNA have been found to be central in vascular processes. Specifically, hsa-miR-146a has been shown to reduce retinal microvascular leakage and improve visual function in diabetic rats, suggesting its potential implication in ocular disease biomarkers development [29]. As a result, in our study, it was our interest to explore their correlation with macular perfusion to elucidate whether perfusion changes over post-operative months are influenced by miRNAs expression profile at the time of surgery and to understand if regulating miRNAs influences visual outcomes over a long period.

Our patients showed good reperfusion at the six-month follow-up, with a significant increase in VD and improvement in BCVA in both conditions.

Upregulation of hsa-miR-144-3p levels was found in vitreous compared to serum specimens in the two groups, while a negative correlation between the absolute variation of values for the inferior sector of the SCP was present in VMD group. Following retinal detachment, the inner retina remains perfused. However, within minutes, the outer retina becomes ischemic with a consequent breakdown of the blood-retinal barrier in the inner retina, probably caused by the diffusion of hypoxic products from the outer retina [30]. The onset of the retinal degeneration process has been related to the hsa-miR-144-3p, which can be considered an antioxidant response regulator. Its upregulation in the presence of oxidative pathway dysregulation and its inverse correlation with the NFR2 activity in retina oxidative stress represents one of the primary key factors for degenerative disease development and inflammation [31].

Besides, a recent study has reported Long Noncoding RNA MIAT to be central in regulating Chronic Retinal Ischemia-Reperfusion damage in rats via the microRNA-203-3p/SNAI2 Axis, suggesting MIAT as a possible therapeutic target for the Retinal ischemia-reperfusion injury [32].

Our study found additional correlations for hsa-miR-320a, an upregulated miRNA found in the two biological specimens. These miRNAs present different correlations with perfusion in the two groups. In fact, in the RRD group, it correlates with a negative variation of SCP, whereas in VMD, it correlates with a positive variation in SCP. The different correlation points out important considerations when dealing with biomarkers, highlighting their role as differential tools in the clinical practice. Moreover, this could potentially highlight the different pathological mechanisms underlying the two conditions.

In detail, hsa-miR-320a can regulate Aquaporin-4 (AQP4) expression and internalization to relieve the edema of Müller cells under the pathological retinal hypoxia stress, thus attenuating their damage. Studies have shown that hypoxia, high intraocular pressure, and reperfusion injury can induce the abnormal expression of AQP4 in Müller cells, resulting in their swelling [33].

Interestingly, these miRNAs showed a significant correlation with final perfusion parameters, indicating a possible role as a regulator of the mechanism leading to reperfusion and possibly better surgical outcomes. Analyzing hsa-miR-451a, we found a slight reduction in our patients. It has been reported to play a role as a regulator gene of cell migration and proliferation in retinal pigment epithelium cells, based on the ability to regulate the mitochondrial membrane permeability and the oxidative stress mediator’s production. In addition, decreased hsa-miR-451a levels were found to be related to neurodegeneration, considering serum specimens. In our work, this data can reflect initial neuronal disruption [34]. Considering the key role of Hsa-miR-451a as an anti-inflammatory miRNA, in our analysis it was downregulated in both disease conditions. In contrast, the other miRNAs are all upregulated, reflecting their involvement in cellular alterations such as inflammation, stress response, and proliferation.

Besides, the hsa-miR-144-3p and hsa-miR-320a may be potential modulators in RRD and VMD, promoting retinal macular reperfusion after the release of macular traction forces after vitrectomy and being involved in the microvessel recanalization.

The mechanisms of regulation and mutual influences of various miRNAs are complex and still need to be completely elucidated. To our knowledge, this is the first report to study altered miRNA expression profiles by correlating them with perfusion analysis over a 6-month follow-up, showing a correlation with vascular changes in patients with vitreoretinal diseases. However, this study has some limitations. Although the prospective nature of our observational case study, the number of enrolled patients could be further increased. Moreover, the choice of using patients’ serum as control must be assessed due to the unfeasibility of vitreous, which was guided by ethical concerns. Furthermore, its potential use as a future biomarker made this choice more suitable, as indicated by previous papers using serum miRNA as indicators of retinal changes [35–37]. Nevertheless, we acknowledge this may represent a key limitation in our study.

Given the relatively small sample size, our results should be interpreted as preliminary and exploratory. Further large-scale, multicentric studies are required to validate these findings and to confirm the potential of miRNAs as reliable biomarkers in vitreoretinal diseases.

Moreover, other factors such as the lack of functional validation of miRNAs and potential confounding factors not considered in the present analysis (e.g., effect of age and gender on miRNA expression) should be acknowledged and considered, representing the limitations in our analysis. However, since a single miRNA may influence the expression of a cluster of genes and an entire pathway, moderating miRNA profiling changes may significantly impact pathogenesis and complications, suggesting a potential role for epigenetics in retinal diseases; thus, further studies are needed to determine the profile of miRNAs in the vitreous of eyes with cytokine expression, to give a more decisive role to miRNAs as biomarkers.

## Electronic supplementary material

Below is the link to the electronic supplementary material.

Supplementary material 1

Supplementary material 2

## Data Availability

The datasets used and/or analysed during the current study are available from the corresponding author on reasonable request.
