# Supplementary material for: MicroRNA profiling in serum and humor vitreous of patients affected by vitreoretinal diseases
Source: Int J Retina Vitreous. 2025 Dec 23;12:14. doi: 10.1186/s40942-025-00765-3 (PMC12836766; doi:10.1186/s40942-025-00765-3)
Supplement: Supplementary file 1 — Supplementary material 1 [file 40942_2025_765_MOESM1_ESM.docx]

**Supplementary Table S1**  MiRNA biological function and related pathway.

|  | **Biological function** | **Pathway** | **Reference** |
| --- | --- | --- | --- |
| Hsa-miR-21-5p | - **Angiogenesis** - **Inflammation** - **Apoptosis** | - PI3K/Akt/FOXO1 - **MAPK,** TGF‑β/SMAD. | 1,2,3 |
| Hsa-miR-144-3p | - Proliferation - Angiogenesis | - NRF2 - MAPK - FoxO | 4, 5 |
| Hsa-miR-146a-5p | - Inflammatory - Cell damage protection - Oxidative stress regulation | - NF-κB | 6, 7 |
| Hsa-miR-222-3p | - **Angiogenesis** - **Cell Proliferation and Migration** | - **PI3K/Akt/FOXO1** - **STAT5A** | 8,9 |
| Hsa-miR-451a | - **Proliferation and** - **Migration** - **Mitochondrial Function** | - **mTOR/HIF-1α/VEGF**. - **PI3K/Akt/Snail**. - **LKB1/AMPK** | 10,11 |
| Hsa-miR-320 | - **Cellular activation** - **Metabolic stress** - **Angiogenesis** | - **mTOR/HIF-1α** - **ERK1/2** | 12,13 |

References:

1. Yan X, Liu Y, Kong X, Ji J, Zhu H, Zhang Z, Fu T, Yang J, Zhang Z, Liu F, Gu Z. MicroRNA-21-5p are involved in apoptosis and invasion of fibroblast-like synoviocytes through PTEN/PI3K/AKT signal. Cytotechnology. 2019 Feb;71(1):317-328. doi: 10.1007/s10616-018-0288-3. Epub 2019 Jan 1. PMID: 30599075; PMCID: PMC6368525.;
2. Aggio-Bruce R, Schumann U, Cioanca AV, Chen FK, McLenachan S, Heath Jeffery RC, Das S, Natoli R. Serum miRNA modulations indicate changes in retinal morphology. Front Mol Neurosci. 2023 Mar 3;16:1130249. doi: 10.3389/fnmol.2023.1130249. PMID: 36937046; PMCID: PMC10020626.)
3. Major JL, Bagchi RA, Pires da Silva J. Application of microRNA Database Mining in Biomarker Discovery and Identification of Therapeutic Targets for Complex Disease. Methods Protoc. 2020 Dec 30;4(1):5. doi: 10.3390/mps4010005. PMID: 33396619; PMCID: PMC7838776.
4. Jadeja, R. N., Jones, M. A., Abdelrahman, A. A., Powell, F. L., Thounaojam, M. C., Gutsaeva, D., Bartoli, M., & Martin, P. M. (2020). Inhibiting microRNA-144 potentiates Nrf2-dependent antioxidant signaling in RPE and protects against oxidative stress-induced outer retinal degeneration. *Redox biology*, *28*, 101336. <https://doi.org/10.1016/j.redox.2019.101336>.
5. Wei, S., Liao, D., & Hu, J. (2024). Inhibition of miR-144-3p/FOXO1 Attenuates Diabetic Keratopathy Via Modulating Autophagy and Apoptosis. *Investigative ophthalmology & visual science*, *65*(1), 1. <https://doi.org/10.1167/iovs.65.1.1>.
6. Kutty RK, Nagineni CN, Samuel W, Vijayasarathy C, Jaworski C, Duncan T, Cameron JE, Flemington EK, Hooks JJ, Redmond TM. Differential regulation of microRNA-146a and microRNA-146b-5p in human retinal pigment epithelial cells by interleukin-1β, tumor necrosis factor-α, and interferon-γ. Mol Vis. 2013 Apr 3;19:737-50. PMID: 23592910; PMCID: PMC3626297.
7. Barutta, F., Corbetta, B., Bellini, S., Guarrera, S., Matullo, G., Scandella, M., Schalkwijk, C., Stehouwer, C. D., Chaturvedi, N., Soedamah-Muthu, S. S., Durazzo, M., & Gruden, G. (2021). MicroRNA 146a is associated with diabetic complications in type 1 diabetic patients from the EURODIAB PCS. *Journal of translational medicine*, *19*(1), 475. <https://doi.org/10.1186/s12967-021-03142-4>
8. <https://www.ncbi.nlm.nih.gov/gene/407007#:~:text=miR%2D222%20acts%20as%20an,miRNA%2C%20by%20controlling%20STAT5A%20expression.&text=Increased%20expression%20of%20MIR222%20is,regulation%20of%20common%20gene%20expression>.
9. Wu, F., Yang, Z., & Li, G. (2009). Role of specific microRNAs for endothelial function and angiogenesis. *Biochemical and biophysical research communications*, *386*(4), 549–553. <https://doi.org/10.1016/j.bbrc.2009.06.075>.
10. Shao, Y., Dong, L. J., Takahashi, Y., Chen, J., Liu, X., Chen, Q., Ma, J. X., & Li, X. R. (2019). miRNA-451a regulates RPE function through promoting mitochondrial function in proliferative diabetic retinopathy. *American journal of physiology. Endocrinology and metabolism*, *316*(3), E443–E452. https://doi.org/10.1152/ajpendo.00360.2018
11. Wu HL, Shao Y, Chen ZN, Zhang H, Zhang XM, Li XR. miRNA-451 regulates rhesus choroid-retinal endothelial cell function and proteome profile. Int J Ophthalmol. 2022 Jun 18;15(6):894-904. doi: 10.18240/ijo.2022.06.06. PMID: 35814901; PMCID: PMC9203463.
12. Liu, S., Yi, J., Lu, S., Cao, J., Xie, B., Tan, Y., & Xiong, W. (2025). The miR-320a/PRDX3 Axis Alleviates the Oxidative Stress and Fibrotic Alterations in Fibroblasts in Thyroid Eye Disease. *Investigative ophthalmology & visual science*, *66*(9), 41. <https://doi.org/10.1167/iovs.66.9.41>
13. Kong L, Sun Y, Chen M, Dai Y, Liu Z. Downregulation of microRNA-320a inhibits proliferation and induces apoptosis of retinoblastoma cells via targeting TUSC3. Exp Ther Med. 2020 Nov;20(5):9. doi: 10.3892/etm.2020.9137. Epub 2020 Aug 25. PMID: 32934674; PMCID: PMC7471862.
